# Tooth caries classification with quantitative light-induced fluorescence (QLF) images using convolutional neural network for permanent teeth in vivo

**DOI:** 10.1186/s12903-023-03669-6

**Published:** 2023-12-08

**Authors:** Eun Young Park, Sungmoon Jeong, Sohee Kang, Jungrae Cho, Ju-Yeon Cho, Eun-Kyong Kim

**Affiliations:** 1 Department of Dentistry, College of Medicine, Yeungnam University, Daegu, South Korea; 2 Research Center for Artificial Intelligence in Medicine, Kyungpook National University Hospital, Daegu, South Korea; 3 Department of Medical Informatics, School of Medicine, Kyungpook National University, Daegu, South Korea; 4 Department of Dentistry, Dongsan Hospital, Keimyung University School of Medicine, Daegu, South Korea; 5 Department of Dental Hygiene, College of Science and Technology, Kyungpook National University, Sangju, South Korea; 6 2559 Gyeongsangde-ro, Sangju, Gyeongsangbuk-do South Korea

**Keywords:** Artificial intelligence, Convolutional neural network, Deep learning, Quantitative light-induced fluorescence, Tooth surface segmentation

## Abstract

**Background:**

Owing to the remarkable advancements of artificial intelligence (AI) applications, AI-based detection of dental caries is continuously improving. We evaluated the efficacy of the detection of dental caries with quantitative light-induced fluorescence (QLF) images using a convolutional neural network (CNN) model.

**Methods:**

Overall, 2814 QLF intraoral images were obtained from 606 participants at a dental clinic using Qraypen C® (QC, AIOBIO, Seoul, Republic of Korea) from October 2020 to October 2022. These images included all the types of permanent teeth of which surfaces were smooth or occlusal. Dataset were randomly assigned to the training (56.0%), validation (14.0%), and test (30.0%) subsets of the dataset for caries classification. Moreover, masked images for teeth area were manually prepared to evaluate the segmentation efficacy. To compare diagnostic performance for caries classification according to the types of teeth, the dataset was further classified into the premolar (1,143 images) and molar (1,441 images) groups. As the CNN model, Xception was applied.

**Results:**

Using the original QLF images, the performance of the classification algorithm was relatively good showing 83.2% of accuracy, 85.6% of precision, and 86.9% of sensitivity. After applying the segmentation process for the tooth area, all the performance indics including 85.6% of accuracy, 88.9% of precision, and 86.9% of sensitivity were improved. However, the performance indices of each type of teeth (both premolar and molar) were similar to those for all teeth.

**Conclusion:**

The application of AI to QLF images for caries classification demonstrated a good performance regardless of teeth type among posterior teeth. Additionally, tooth area segmentation through background elimination from QLF images exhibited a better performance.

## Background

Dental caries is recognized as one of the most common oral diseases worldwide, affecting people throughout their lives. If not treated properly, it can cause tooth pain and even tooth loss [1]. However, because caries is often insidious in the early stage, many patients tend to visit dental clinics when it has already reached the advanced stage. Notably, advanced-stage caries can place a biological, social, and financial burden on both individuals and the nation [2]. Thus, early detection of dental caries can prevent its progression and help improve oral health, which is also related to overall health and quality of life [3]. Various methods have been attempted by dentists for accurate, easy, and quick detection of dental caries using new technologies and oral examination.

Conventional oral examination is primarily performed via visual inspection and tactile sensation by dentists, which are the most effective methods for caries detection in the majority of cases. However, in some cases, objective detection is needed, for example, in obscured, noncavitated, initial lesions of caries [4], in order to overcome differences between examiners due to the subjective evaluation. Thus, various optical technologies have been developed for detecting and quantifying carious lesions [5].

One of these optical techniques is quantitative light-induced fluorescence (QLF). It involves the use of a visible light with a wavelength of 405 nm, which falls in the blue region of the spectrum. This technique is based on the principle that the mineral content of the tooth changes the autofluorescence of the specific lesion in the tooth. Moreover, the demineralization of enamel in carious lesion reduces autofluorescence and causes darkening of the enamel [6]. In the relevant literature, QLF has been proven to be useful in the early detection, quantification, and monitoring of noncavitated carious lesions [7, 8]. As QLF images can be visualized, stored, and transmitted in the form of digital files using proper digital equipment, they can be processed using an artificial intelligence (AI) model. At present, AI is widely used for the detection of various diseases. Moreover, convolutional neural network (CNN)—a type of AI model—has demonstrated excellent performance in computer vision and has been commonly used to analyze visual imagery [9]. In the field of dentistry, several studies applied CNNs to detect carious lesions, and these CNNs exhibited a relatively good performance on various types of images, such as periapical radiographs [10, 11], bitewing radiographs [12–14], panoramic radiographs [15], radiovisiography images [16], oral photographs [17–19] and their masked images [20], and near-infrared transillumination images [21, 22]. Specially, in case of oral photographs, there were some previous studies showing favorable performance of AI model to detect caries using white light images which were captured by professional single-reflex lens camera, intraoral camera (IOC), or smartphone [17–19]. Compared to these white light images, QLF images could more eminently visualize demineralization of enamel and existence of cariogenic bacteria, which contribute to recognize the caries easily through visual examination [6–8]. Considering these characteristics, application of CNN model to QLF images was inferred to yield favorable performance for screenning of teeth caries.

However, in the literature, studies applying QLF images to CNNs for caries detection are limited; however, several studies have reported the use of AI models for detecting plaque and detection of gingivitis [23–26]. With the development of technology, AI-based automated detection of dental caries seems promising for rapid and accurate detection of caries, which may further reduce the tasks of dental staff and enable the provision of dental care to a larger population at a lower cost. Therefore, we evaluated the performance of the QLF image-based CNN model for dental caries detection according to the type of tooth and the presence of manual segmentation to demonstrate the potential of using QLF image in the field of AI.

## Methods

### Study protocol and dataset

This study included 606 patients who visited the dental clinic of universal hospitals for oral examination. In this study, Qraypen C® (QC, AIOBIO, Seoul, Republic of Korea) was used to collect 2814 intra oral pictures as the dataset from October 2020 to October 2022. As a registered diagnostic medical instrument in Korea, this device is used in general dental clinics to detect caries by capturing QLF image of the intraoral cavity. The study protocol was approved by the Institutional Review Board (IRB) of the University and the study was performed in accordance with the principles of the Declaration of Helsinki. The participants who had at least one permanent tooth without dental crown restoration were recruited after providing information according to the protocol. However, those who did not have ability to cooperate to capture QLF images or did not provide informed written consents were excluded. Moreover, this study was conducted in accordance with the guidelines of the Standards for Reporting of Diagnostic Accuracy Studies [27]. After the examination of caries by three dentists under dental light with air syringe in the dental chair, QLF images with a resolution of 1,280 × 720 pixels were captured for validation test of caries teeth classification with CNN model. There were no limitations related to the conditions of teeth or caries severity in the dataset, such as the amount of saliva and the presence of restorations, pit and fissure stain, orthodontic wire, tooth wear, or carious cavity formation. Finally, both permanent anterior (230 images) and posterior (2,584 images) teeth were included in the whole dataset regardless of tooth surface (occlusal surface: 2413, smooth surface: 401). Moreover, each QLF image was manually segmented into teeth for background noise reduction. Details of the segmentation process are explained in the next section.

### Annotation for caries and dataset assignment

Each QLF image was annotated in terms of the presence or absence of carious teeth by examining the white light image which was captured at the same teeth sequentially by Qraypen C® according to the International Caries Detection and Assessment System (ICDAS) considering each clinical chart record as the reference. For consistency, one dentist who was trained using documents on ICDAS caries visual detection criteria downloaded at home page of ICCMSTM (https://www.iccms-web.com/) [28] examined whole images and labelled as caries when code was 3, 4, 5 or 6 according to ICDAS criteria (Code 3: Localized enamel breakdown, Code 4: underlying dark shadow from dentin, Code 5: Distinct cavity with visible dentin, Code 6: Extensive distinct cavity with visible dentin).

Furthermore, the tooth surfaces in each QLF image were segmented by pixelwise labeling using a tagging tool (https://supervise.ly) by one dental hygienist. The entire dataset comprised 2,814 intraoral QLF images (including 1,701 images of carious teeth and 1,113 of sound teeth) of 606 patients. In terms of the type of tooth, the entire dataset comprised 1,441, 1,143, and 230 images of molar, premolar, and anterior teeth, respectively.

The dataset was further divided into three subsets: training (1,575 images), validation (394 images), and test (845 images). QLF images corresponding to 30% of the entire dataset were first randomly assigned to the test dataset, considering the ratio of carious teeth to sound teeth programmatically using scikit-learn, a python library. Subsequently, the remaining images were randomly divided into the training and evaluation subsets at a ratio of 4:1 during training. Moreover, for model training according to the type of tooth (molar or premolar), the same protocol was applied to assign each dataset (molar, 1,441 images; premolar, 1,143 images) to the train, validation, and test subsets. Detailed information of the dataset is presented in Table 1. Furthermore, the QLF dataset of 2,709 images was masked by a manual teeth segmentation map with 1,623 and 1,086 images of carious teeth and sound teeth, respectively (train, 1,516 images; validation, 380 images; and test, 813 images). Notably, the masked images were prepared by manually eliminating the background, excluding the tooth surface, from each QLF image. Notably, the QLF images included in the test subset were never used as data for the train or validation subset during the process of model training for the objective evaluation of the CNN model.

**Table 1:** Dataset composition of quantitative light-induced fluorescence images

Subset			All teeth			Premolar			Molar		
	Total	(%)		Caries	Normal	Total	Caries	Normal	Total	Caries	Normal
Train	1,575	(56.0)		961	614	640	308	332	806	552	254
Validation	394	(14.0)		229	165	160	85	75	202	144	58
Test	845	(30.0)		511	334	343	169	174	433	299	134
Total	2,814	(100)		1,701	1,113	1,143	562	581	1,441	995	446

### Deep learning algorithm applications for caries classification

The development of deep learning algorithms as a visual inspection tool has prompted researchers to evaluate various CNN architectures or component blocks of these architectures. Inception [29] is one of the novel components of CNN aimed at enhancing feature extraction in the network. However, various convolutional branches of this component lead to additional computational costs compared with the simple serial convolutional layers. Xception, a novel deep CNN architecture with refactored and generalized version of Inception, was selected for classification of QLF images [30] owing to its computational efficiency and good performance. Although Xception used the same number of parameters as the original Inception V3, it demonstrated better performance than Inception V3 in the classification of various images. Moreover, the model was trained to classify caries on images by fine-tuning pretrained weights from the ImageNet dataset. Furthermore, image augmentation (such as horizontal or vertical flipping) or rotation, brightness and contrast adjustment, or image blurring was randomly applied to prevent overfitting in the training phase. Moreover, original images were resized to 612 × 420 pixels for fast inference of the classification network. To apply k-fold cross-validation, training was performed five times by sequentially replacing the validation sets at a 1:5 ratio. Each training was performed in a range of 15–20 epochs. Early stopping was done in case of increasement of the validation loss. The parameters were updated using binary cross-entropy loss and the Adam optimizer. The initial learning rate was set at 0.00001, and a dynamic learning scheduler was applied using the ramp-up and step-down decay method suggested by Chris Deotte (code shown at https://www.kaggle.com/code/tuckerarrants/kfold-efficientnet-augmentation-s). The batch size was set as large as possible as the memory allowed. The details of information on implementation were obtained using Google CoLab Pro—a type of cloud service for deep learning research. Notably, NVIDIA A100-SXM4-40GB GPU and Intel Xeon CPU were used for implementation, and Python 3.3.10, TensorFlow 2.9.2, and CUDA 11.2 were used as deep learning frameworks.

### Statistical analysis

To evaluate the classification performance of the CNN model, the sensitivity, specificity, negative predictive value (NPV), f1-score, precision, accuracy, and area under the receiver operating characteristic curve (AUC) were calculated according to the segmentation of the tooth surface or types of tooth (premolar and molar), respectively. Finally, the receiver operating characteristic (ROC) curve was drawn.

## Results

Among a total of 606 participants, 542 ones allowed to provide their demographic information such as age and gender, in which mean age was 50.1(± 18.3) years and male and female was 290 and 252, respectively. The performance indices of the CNN model for caries classification using the test subset of 845 images (carious teeth, 511 images; sound teeth, 334 images) are presented in Table 2. Most indices were found to be > 80%, except for specificity and NPV, which were 77.5% and 79.5%, respectively. For example, sensitivity, f1-score, and AUC were 86.9%, 86.2%, and 90.9%, respectively, indicating a relatively good performance. In particular, performance was improved using a test dataset of 813 segmented images for the tooth surface by manually eliminating the background, indicating that all indices were > 80%, including f1-score, 87.9%; precision, 88.9%; and AUC 92.8% (Table 2).

**Table 2:** Evaluation measures for caries classification according to tooth area segmentation

Evaluation metrics	Segmentation for teeth area	
	No	Yes
True-positive case, n (%)	444 (52.5)	423 (52.0)
False-positive case, n (%)	75 (8.9)	53 (6.5)
True-negative case, n (%)	259 (30.7)	273 (33.6)
False-negative case, n (%)	67 (7.9)	64 (7.9)
Sensitivity	0.869	0.869
Specificity	0.775	0.810
Negative predictive value	0.795	0.837
f1-scores	0.862	0.879
Precision	0.856	0.889
Accuracy	0.832	0.856
AUC	0.909	0.928

However, the performance of the CNN model, which used a dataset with only premolar or molar images, was similar to that for all teeth shown in Table 2 (Table 3). When the CNN model was trained and tested using molar images alone, most of the performance indices were > 80%, except for specificity (71.6%) and NPV (72.2%): sensitivity, 87.6%; f1-score, 87.5%; and AUC, 88.1%. However, all performance indices were > 80% in the case of premolar images: sensitivity, 83.4%; accuracy, 83.4%; and AUC 90.5% (Table 3).

**Table 3:** Evaluation metrics for caries classification according to the types of teeth (molar and premolar)

Evaluation metrics	Q-ray images	
	Molar	Premolar
True-positive case, n (%)	262 (60.5)	141 (41.1)
False-positive case, n (%)	38 (8.8)	29 (8.5)
True-negative case, n (%)	96 (22.2)	145 (42.3)
False-negative case, n (%)	37 (8.5)	28 (8.2)
Sensitivity	0.876	0.834
Specificity	0.716	0.829
Negative predictive value	0.722	0.834
f1-scores	0.875	0.832
Precision	0.873	0.829
Accuracy	0.827	0.834
AUC	0.881	0.905

**Fig. 1:**
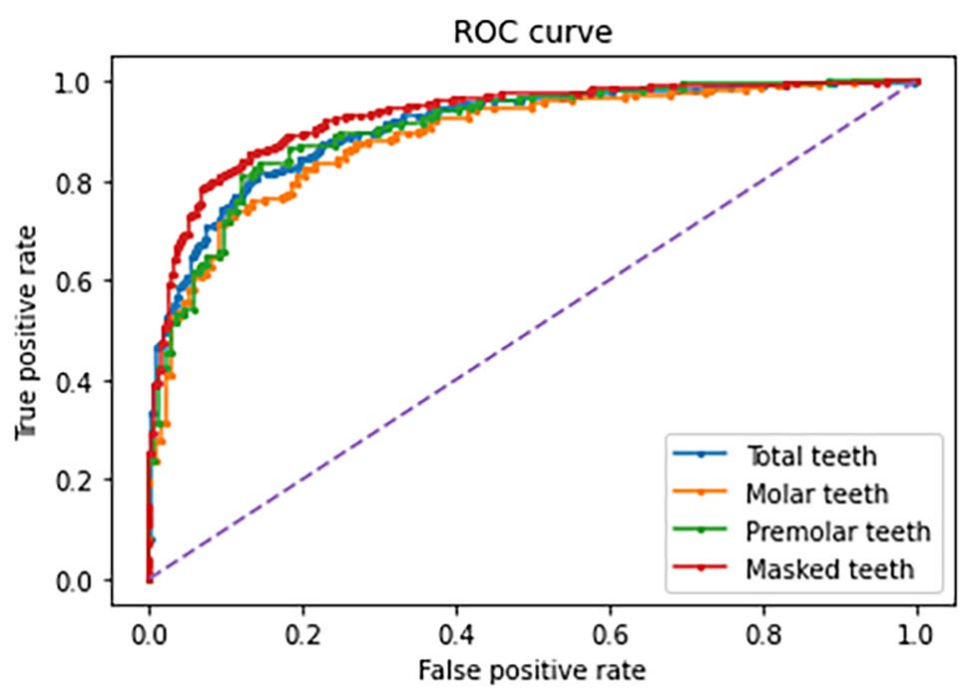
Receiver operating characteristic (ROC) curve according to tooth area segmentation and type of tooth (premolar and molar). The Blue, orange, and green line are ROC curves of artificial intelligence (AI) models trained using total teeth, molar, premolar images without masking background, respectively. The red line is a ROC curve of AI model trained using total teeth images with masking background through tooth area segmentation

**Fig. 2:**
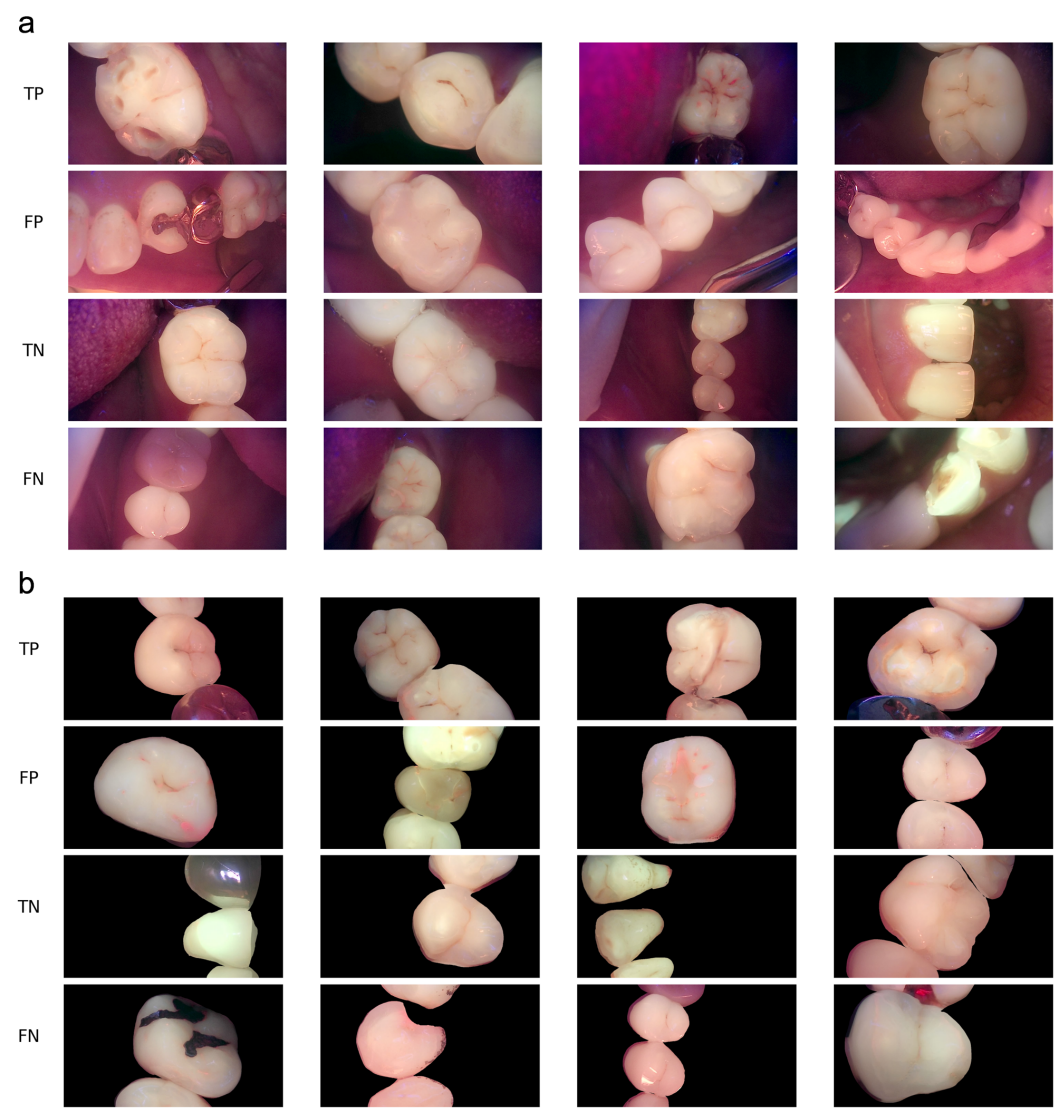
Results of classification using original and masked quantitative light-induced fluorescence (QLF) images (**A**) original QLF images, (**B**) masked QLF images. True positive (TP), false positive (FP), true negative (TN), and false negative (FN) cases were shown, respectively

The ROC curves of each model according to the dataset are shown in Fig. 1, in which the images of all teeth, molars, and premolars as well as the masked images of all teeth were used as the dataset. The best result was obtained in the case of a masked image of all teeth. Four cases of true-positive, false-positive, true-negative, and false-negative results in the test datasets of original QLF and segmented QLF images, respectively, are shown in Fig. 2.

## Discussion

QLF imaging—an effective examination method for detecting dental caries—may be practically applied in clinical dentistry. The development of AI in medical field has prompted the need for evaluating the performance of CNN models using QLF images. Therefore, this study aimed to evaluate the CNN model for caries classification using QLF images, which could be commonly obtained in general dental clinics. The following results were obtained: (1) QLF images could effectively be used in the caries classification task by the CNN model. Further, when masked QLF images, which were manually prepared by eliminating the background of the images, were used, performance was improved. (2) When subset of data according to the tooth type such as premolar or molar was used to train AI model separately, performance indices were little reduced. However, overall performance was close to that of all teeth group. These results indicate that QLF images can be effectively used for the CNN algorithm in caries classification, with comparable performance to the deep learning model using IOC images.

IOC has been widely used in dental clinics as a visual inspection tool for caries detection because it is inexpensive, easy-to-use, and capable of capturing high-quality images [31–34]. Accordingly, some studies have investigated deep learning algorithms using oral photographs for dental caries detection [17, 20], and these algorithms exhibited a relatively good performance. In contrast, our model that used QLF images demonstrated a competitive performance, with most performance indices of > 80% and AUC of 92.8%. Notably, the value of AUC (92.8%) in our study was better than 85.6% [17] and 83.7% [20] in studies with models using IOC images for caries classification. Although the performance indices slightly improved after the background was eliminated [20], the competitive performance of QLF images may be mainly attributed to the specific fluorescence, which can be detected in the QLF system [35].

When a visible blue light of 405 nm is applied at the teeth using a QLF device, an area of ​​carious lesion can be observed, where the autofluorescence decreases due to the loss of minerals in the tooth, resulting in a dark appearance; moreover, red fluorescence can occur due to porphyrin—a metabolite secreted by bacteria in carious lesions. In addition, plaque exhibits red fluorescence owing to the porphyrins in the bacterial biofilms [35]. Therefore, QLF images can be effectively used in dental clinics to detect both dental caries and dental plaques [36, 37]. Among various QLF devices, a pen-type QLF device equipped with a digital camera with a special light source filter and a digital image sensor is suitable to easily capture intraoral images [38]. Further, a captured intraoral QLF image can be digitalized and immediately visualized in a computer monitor. This is a quantifiable method for carious lesions and dental plaques that demonstrates good potential for use in dental clinics. Therefore, applying an AI model to these QLF images may be desirable, which may help improve the prediction of dental caries and plaque; however, further studies are required to validate the same.

This study has several limitations. The first, our dataset was consisted of posterior ones (i.e. molar or premolar) (92%) as tooth type and occlusal ones (86%) as tooth surfaces. Therefore, to get sufficient images including anterior teeth and smooth surface is need for balance of dataset. The second, evaluation of AI model according to caries severity was not performed. Although caries severity according to cavity formation was annotated, these images were so small (13%), which was insufficient for AI model training. Therefore, dataset including sufficient cases according to caries severity is need. The third, existence of caries was annotated only at the whole image level. Caries lesion area was not annotated, which is a limitation of making explainable AI model. In future, through localization of caries lesion in images, it needs to contribute to make explainable AI model. The fourth, exact sample size was not assessed before recruiting participants. However, considering one previous study [12] as a reference, if the accuracy of 0.8 and 0.75 for deep learning model and dentists, standard deviation of 0,4 and a study powered at 1-beta of 0.8 with alpha of 0.05 were assumed, a total of number of teeth needed for test dataset was 442. Based on this, considering design effect (DE) of which formula DE = 1+(m-1)*ICC with m of 5, the cluster size and ICC of 0.2, the Intraclass Correlation Coefficient, test dataset must include 796 teeth. In our dataset, most image were about posterior teeth which were included lower than 5 per image. Therefore, our test dataset (n = 845 images) could be regarded to be sufficient. The last, we performed segmentation manually to avoid error from auto-segmentation by the AI model because we focused on the possibility of using QLF image as a dataset for caries prediction. Therefore, further studies on automatic segmentation models are required for dental field applications.

However, to the best of our knowledge, this is the first study evaluating an AI model to detect caries regardless of its severity using QLF images taken from oral cavity of participants in dental clinic using IOC. This study differs from previous studies in which AI models were used to detect caries from tooth specimen or initial caries only, or identify oral hygiene conditions, such as the presence of plaque [26, 39, 40].

## Conclusions

The results of this study indicate that QLF images can be effectively used as a dataset for AI application to diagnose dental caries. Accordingly, this study may contribute to a comprehensive evaluation of oral health risk factors, including dental caries and plaques, using the QLF system. Thus, further studies on the development of AI model for evaluating oral health and hygiene using QLF images are warranted.

## Data Availability

The datasets generated during the current study are not publicly available due to privacy of participants but are available from the corresponding author on reasonable request.

## Abbreviations

CNN: Convolutional neural networks
QLF: Quantitative light-induced fluorescence
IOC: Intra-oral camera
DL: Deep learning
AI: Artificial intelligence
CAD: Computer-aided diagnostic
DSLR: Digital single-lens reflex
STARD: Standards for Reporting of Diagnostic Accuracy Studies
ICDAS: International Caries Detection and Assessment System
ISVRC: ImageNet Large-Scale Visual Recognition Challenge
RPN: Region proposal network
NPV: Negative predictive value
AUC: Area under the receiver operating characteristic curve
ROC: The receiver operating characteristic
